# Expression Patterns of Muscle-Specific miR-133b and miR-206 Correlate with Nutritional Status and Sarcopenia

**DOI:** 10.3390/nu12020297

**Published:** 2020-01-22

**Authors:** Francesca Iannone, Alberto Montesanto, Erika Cione, Paolina Crocco, Maria Cristina Caroleo, Serena Dato, Giuseppina Rose, Giuseppe Passarino

**Affiliations:** 1 Department of Biology, Ecology and Earth Sciences, University of Calabria, 87036 Rende, Italy; francesca.iannone@unical.it (F.I.); alberto.montesanto@unical.it (A.M.); crocco.paola@gmail.com (P.C.); serena.dato@unical.it (S.D.); giuseppe.passarino@unical.it (G.P.); 2 Department of Pharmacy, Health and Nutritional Sciences, University of Calabria, 87036 Rende, Italy; erika.cione@unical.it (E.C.); mariacristinacaroleo@virgilio.it (M.C.C.)

**Keywords:** myomiRs, miR-133b, miR-206, sarcopenia, muscle wasting, nutritional status, aging

## Abstract

Sarcopenia and malnutrition are commonly occurring conditions in the elderly that frequently coexist, leading to substantial effects on morbidity/mortality. Evidence established muscle-specific microRNAs (miRNAs) or myomiRs as essential regulators of skeletal muscle processes, from myogenesis to muscle homeostasis. This study aimed to evaluate the association between myomiRs and sarcopenia and explore the potential of nutrition in mediating this association. qPCR was employed to characterize the myomiR-1, -133a/b, -206, -208b, and -499 expression profiles of 109 non-sarcopenic and 109 sarcopenic subjects. In our sample, the proportion malnourished or at-risk subjects was higher in sarcopenia (*p* < 0.001). Among the detected myomiRs (miR-133a/b and miR-206), lower levels of miR-133b was significantly associated with the presence of sarcopenia (*p* = 0.006); however, this relationship was not independent from nutritional status in multivariate analysis, suggesting a mediating effect of nutrition on the relationship between miR-133b and sarcopenia. Correlation analyses showed that lower miR-133b levels were associated with poor nutritional status (Mini Nutritional Assessment Long Form (MNA-LF) score, *p* = 0.005); furthermore, correlations with albumin, ferritin, and iron were found. Similar results were obtained for miR-206. Statistically more significant correlations were observed in subjects with sarcopenia. In conclusion, our findings highlight a nutrient-miR-133b/miR-206 pathway having a potential role in the age-related muscle decline.

## 1. Introduction

The wide-ranging variation in the rate and quality of aging results from the intertwined interactions among a variety of genetic/epigenetic and environmental/lifestyle factors that impinge lifelong on our body. Nutrition is seen as one of the most important modifiable lifestyle factors affecting the whole aging process, with evidences increasingly indicating that nutrition is a major risk factor for the onset of chronic conditions [1,2]. Many changes accompanying aging, such as anorexia of aging, body composition changes, worsening of oral health, and decline of sensory functions as well as pathological and socio-environmental factors, can promote a poor nutritional status due to inadequate nutritional intake [3]. Malnutrition represents a common problem in older persons, with data showing that up to 22% of older adults are malnourished and over 45% are at risk of malnutrition [4]. The consequences of malnutrition are diverse, severe, and long lasting. People with a poor nutritional status experience an accelerated transition from vulnerability to frailty and dependence and also are at increased risk of mortality [5,6,7].

The skeletal muscle is an adaptive tissue involved in the global metabolic homeostasis regulation. It is a key site for glucose uptake and storage and the largest reservoir of proteins and free amino acids in the body that plays a crucial role in the global metabolic homeostasis via inter-organ crosstalk [8]. As the largest metabolic organ in the body, skeletal muscle is strongly influenced by the nutritional status. Indeed, malnutrition, together with factors related to age such as chronic inflammation, oxidative stress, and hormonal changes, is a key contributor to development of sarcopenia, the progressive and generalized loss of muscle mass and strength that accompanies aging and the leading cause of disability, morbidity, and mortality in older adults [9,10,11,12]. According to the current estimates, 5%–10% of elderly people aged 60–70 years and 11%–50% of those over the age of 80 are facing this disability [13]. Thus, these statistics prompt this issue as a serious public health concern.

The maintenance of muscle homeostasis is finely regulated by the orchestrate action of muscle-specific transcription factors and epigenetic regulators. These include DNA methylation, histone modification, as well as the non-coding microRNAs (miRNAs) [14]. MicroRNAs are small molecules, approximately 21–22 nucleotides in length, able to regulate the expression of their targets by binding to 5′UTR, coding regions or 3′UTR of mRNAs, causing translational inhibition or mRNA degradation [15]. Because of their versatility (the same miRNAs can target a large number of mRNAs, and the same mRNA can be targeted by many miRNAs), miRNAs exert extensive regulatory control over various biological processes, greatly influencing both physiological and pathological processes [16]. Recently, several miRNAs have been found up- or downregulated in skeletal muscle during aging (reviewed in [17]), suggesting that this differential expression may underlie the reduced age-related muscle functionality [18,19,20,21]. In particular, a pivotal role in almost all aspects of skeletal muscle developments is presently assigned to a group of specific skeletal muscle miRNAs, designated myomiRs. This group comprises miRNA-1, miRNA-133a, miRNA-133b, miRNA-206, miRNA-208b, and miRNA-499, whose characteristic features are shown in Supplementary Materials, Figure S1. They play a crucial role in the context of muscle physiology by targeting genes involved in signaling pathways that regulate muscle skeletal development and growth, differentiation, and regeneration [22,23,24,25,26]. An interesting clue is that many of them can regulate, or be regulated, by components of the IGF-1/Akt/mTOR signaling pathway, known to regulate skeletal muscle protein synthesis (MPS) and muscle protein breakdown (MPB) [27], two processes that are highly responsive to anabolic stimuli, such as physical activity and food intake. Interestingly, myomiRs expression has been shown to be modulated by exercise, although the directionality of these changes appear to be sensitive to exercise type [28,29], and by the combined ingestion of essential amino acid [30,31], proteins [32], and carbohydrates [31]. This is particularly intriguing, considering that the imbalance between MPS and MPB, associated with impaired rate of muscle anabolic responses to exercise and nutrients (in particular protein intake), termed “anabolic resistance”, may underpin the progression of sarcopenia [33]. Moreover, the study by Drummond and colleagues [30] also examined myomiR expression patterns in muscle in relation to aging, detecting no differences in basal expression of mature functional myomiRs between elderly and younger men. Nevertheless, the expression of myomiRs in response to resistance exercise and ingestion of essential amino acids was different between elderly and young. However, contrasting results were found by Nielsen and colleagues [34], who reported higher expression of miR-1 and 133a/b in older men compared to young men.

As far as we know, there are no studies evaluating circulating myomiR levels in sarcopenic subjects and the possible relationship with nutrition. Thus, the main objective of this study was twofold: firstly, to examine whether expression levels of myomiRs transcripts in old individuals are associated with sarcopenia; and secondly, to explore whether the associations are influenced by nutritional status. This study, by identifying those myomiRs changing their expression in sarcopenic subjects, may provide new useful biomarkers of sarcopenia. Furthermore, the potential relationship with nutritional status could provide insights in the molecular pathways underpinning sarcopenia and help to develop therapeutic targets or set up lifestyle interventions to prevent or delay the onset of the age-related muscle decline.

## 2. Materials and Methods

### 2.1. Participants

Participants were recruited from elderly nursing homes located in the province of Crotone and Cosenza in the Calabria region (southern Italy), as part of a study carried out for monitoring the quality of aging in the whole region. Subjects were eligible to participate in the study if they were older than 65 years of age and of Calabrian ancestry. We excluded patients with cardiac involvement and severe neuropsychiatric illness that caused patients to be unable to understand and perform instructions and to provide written informed consent. In total, 218 subjects were enrolled, 79 were males and 139 females with a mean age of 81.6 (±7.10) years. At recruitment, eligible and consenting participants were subjected to a multidimensional geriatric assessment including information on demographics (age, sex, education), cognitive status, functional abilities, and physical health, obtained through a structured questionnaire administered during an interview with a trained operator. A peripheral blood sample was collected from each participant for clinical and laboratory examinations.

### 2.2. Ethics Statement

All subjects gave their informed consent for inclusion before they participated in the study. The study was conducted in accordance with the Declaration of Helsinki, and the protocol was approved by the local Ethical Committee (Comitato Etico Regione Calabria-Sezione Area Nord) on 2017-10-31 (code n. 25/2017).

### 2.3. Assessment of Muscle Mass

Muscle mass was measured by bioelectrical impedance analysis (BIA) using a Quantum/S Bioelectrical Body Composition Analyzer (AkernSrl, Florence, Italy). Whole-body BIA measurements were taken between the right wrist and ankle with subject in a supine position. Muscle mass was calculated using the BIA equation of Janssen and colleagues [35]: Skeletal muscle mass (kg) = ([height^2^/BIA resistance × 0.401] + [gender × 3.825] − [age × 0.071]) + 5.102, where height is measured in centimeters; bioelectrical impedance analyses resistance is measured in ohms; for gender, men = 1 and women = 0; age is measured in years. Absolute muscle mass was converted to skeletal muscle index (SMI) by dividing the value by the square of the height in meters (kg/m^2^).

### 2.4. Measurement of Muscle Strength

Muscle strength was assessed as hand grip strength (HGS) using a handheld dynamometer (SMEDLEY’s dynamometer TTM) while the subject was sitting with the arm close to his/her body. The test was repeated three times with the stronger hand and the maximum of these values was considered.

### 2.5. Assessment of Muscle Performance

Gait speed was measured using the 4 meter (m) walking test. Patients were asked to walk straight for 4 m at their usual speed for the measurement of a 4 m walk time. Timing began when subjects initiated foot movement and stopped when one foot contacted the ground after completely crossing the 4 m mark. Gait speed (m/s) was calculated by dividing the distance covered by the 4 m walk time (s). The best time of two attempts was recorded.

### 2.6. Evaluation of Disability

The management of activities of daily living or ADL (bathing, dressing, toileting, transfer from bed to chair, and feeding) was assessed using a modification of the Katz Index of ADL [36]. The assessment was based on what the subject was able to do at the time of the visit. The score is given counting the number of activities in which the participant is dependent or independent at the time of the visit. For the analyses, ADL scores were dichotomized as one if the subject was not independent in all five items and zero otherwise.

### 2.7. Nutritional Assessment

We used the Mini Nutritional Assessment (MNA) to assess the nutritional state of the participants [37]. The short form of the MNA (MNA-SF) includes six queries regarding food intake, weight loss, mobility, psychological stress, or acute disease, the presence of dementia or depression, and body mass index (BMI). For this screening tool, the maximum score is equal to 14. A score ≥12 indicates that the subject has an acceptable nutritional status, whereas a score <12 indicates risk of malnutrition; in this last case it was then necessary to complete the MNA Long Form (MNA-LF). The MNA-LF consists of 18 items from four sections: global evaluation, anthropometric assessment, dietetic assessment (including number of full meals, fruit/vegetables, and water consumption), and self-assessment. The total score ranges from 0 to 30. Individuals were considered malnourished if they scored <17, at risk for malnutrition if they scored between 17 and 23.5, and well-nourished if their scores were ≥24.

Anthropometrics and biochemical markers of nutritional status reported in Table 1 were measured using standard laboratory procedures in all the subjects.

### 2.8. Diagnosis of Sarcopenia

Sarcopenia was diagnosed by measuring muscle strength, muscle mass, and physical performance according to the revised criteria suggested by the European Working Group on Sarcopenia in Older People (EWGSOP2) [38]. Sarcopenia was defined as low muscle strength (HGS <27 kg in males; <16 kg in females) associated with either low skeletal muscle mass index (SMI; <8.50 kg/m^2^ in males; <5.75 kg/m^2^ in females) or low gait speed (<0.8 m/s).

### 2.9. Blood Plasma Collection

Blood plasma samples were prepared as follows. Venous blood samples were drawn after a 12 h overnight fast and processed within 2 h from collection. Plasma for miRNAs analysis was separated by centrifugation at 1800 g for 10 min at room temperature, collected in RNase-free tubes and further centrifuged at 1200× *g* for 20 min at 10 °C to completely remove contaminant cells. Finally, plasma samples were divided in aliquots to avoid freeze–thaw cycles and finally stored at −80 °C up to the RNA extraction.

### 2.10. RNA Extraction and miRNA Quantification

MicroRNAs were isolated from 200 μL of plasma using miRNeasy*^®^* Serum/Plasma kit (Qiagen, Hilden, Germany) according to the manufacturer’s instructions. Five volumes of QIAzol Lysis Reagent were added to each sample. After 5 min incubation at room temperature, 200 μL of chloroform was added together with 3.5 μL of *Arabidopsis thaliana* miR-159a (assay ID 000338) as a spike-in control and the mixture was incubated for 3 min. The lysate was separated into aqueous and organic phases by centrifugation for 15 min at 12,000× *g*. Next, 1.5 volumes of 100% ethanol were added to the aqueous phase and the solution was passed through the RNeasy MinElute spin column in order to make the small RNAs tie to the membrane. Using appropriate washing buffers, phenol and other contaminants were expelled. Finally, RNAs was washed with 80% ethanol and eluted with 14 μL of RNase-free water. RNA yield was quantified on the Qubit 2.0 Fluorometer (Life Technologies, Milan, Italy) and it was around 30–50 ng/mL each sample. Of this, 5 μL was converted in cDNA using TaqMan*^®^* microRNA Reverse Transcription Kit (Life Technologies) and stem–loop specific RT primers for each selected human miRNA (hsa-miR-1 assay, ID 002222; hsa-miR-133a, assay ID 002246; hsa-miR133b, assay ID 002247; hsa-miR-206, assay ID 000510; hsa-miR-208b, assay ID 002290; hsa-miR-499, assay ID 001045). Small nuclear (snRNA) U6 was used as endogenous control (assay ID 001973). The mixture was incubated at 16 °C for 30 min, 42 °C for 30 min, and 85 °C for 5 min. Afterward, quantitative real-time PCR was performed on a QuantStudio3™ Real-Time PCR System (Applied Biosystems, Milan, Italy) with automatic baseline setting, using TaqMan*^®^* Universal Master Mix 2x. without uracil-N-glycoslyase (UNG) (Applied Biosystems), 1 μL 20x Taqman miR Assay (Life Technologies) and 1.33 μL RT product. Real-time reaction was carried out at 95 °C for 10 min, followed by 40 cycles of 95 °C for 15 s and 60 °C for 60 s. All reactions, including the no-template controls, were run in triplicate. The relative expression levels of each miRNA in comparison with the normalizer were then calculated using the comparative threshold (Ct) method 2^−ΔCt^ [39], where ΔCt represents the difference between each miRNA and the normalizer (average Ct for the miRNA minus average Ct for snRNA U6). Expression levels (2^−ΔCt^) were log-transformed to better fit a normal distribution.

### 2.11. Statistical Analysis

Continuous variables are presented as means and standard deviations (SD), while categorical variables are presented as percentages. Shapiro–Wilk test was used to assess the normality assumption for continuous variable. In case of violation, suitable data transformation methods were adopted for addressing non-normality. Continuous and categorical variables were compared between sarcopenic and non-sarcopenic subjects using independent sample t-test or chi-square test as appropriate. A binary logistic regression analysis was used to assess the association between sarcopenia and the variability of the assessed plasma miRNA levels. Multivariate analysis was made after adjustment for variables that were significantly different between sarcopenic and non-sarcopenic subjects, with the exception of those strictly related to sarcopenia diagnosis (hand grip strength, skeletal muscle index, and gait speed). Correlation analyses were performed using Spearman’s correlation coefficient to determine the magnitude of association between plasma miRNA levels and MNA scores, as well as biochemical markers of nutritional status. Statistical significance was defined as two-tailed *p*-value <0.05. All statistical analyses were performed using IBM SPSS statistics for Windows v25 (IBM Corp., Armonk, NY ).

## 3. Results

We enrolled a total of 218 subjects with a mean age of 81.6 years of whom 109 (50.0%) were identified as affected by sarcopenia according to the EWGSOP2 criteria. Table 1 shows the baseline characteristics of the study participants, stratified by the presence of sarcopenia. Individuals with sarcopenia were older compared to those without the condition.

Compared with non-sarcopenic subjects, dependency in ADL was more prevalent in sarcopenic subjects (43.7% vs. 75.9%, *p* < 0.001). Subjects with sarcopenia who were malnourished according to MNA-SF were 59.4%, whereas only the 35.4% of the non-sarcopenic subjects were classified as having a malnutrition status (*p* = 0.002). After the complete assessment of MNA (MNA-LF), the proportion of subjects malnourished and at risk of malnutrition remained significantly higher in the sarcopenic group than in the non-sarcopenic group (77.4% vs. 50.0% *p* = 0.002). Moreover, significant differences in several clinical–biochemical parameters were observed between the two groups of subjects (Table 1).

Plasma levels of the myomiRs were investigated in the cohort studied. First, in order to verify the reliability of snRNA U6 as endogenous control to standardize miRNA expression, Ct values of U6 were compared between sarcopenic and non-sarcopenic groups. No significant difference was observed (*p* = 0.528), suggesting that U6 is constitutively expressed in plasma regardless of disease condition, and thus supporting its use as a reliable normalization control. Out of the six miRNAs we analyzed, miR-1, miR-208b, and miR-499 were undetected in this sample, while miR-206, miR-133a. and miR-133b were found in all subjects.

Univariate analysis ruled out the correlation between age/gender and myomiRs levels (*p* > 0.05).

**Figure 1:**
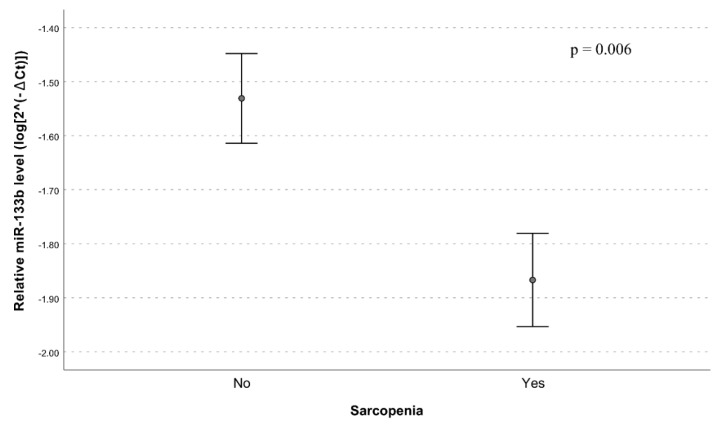
Relative miR-133b expression in plasma from sarcopenic and non-sarcopenic subjects. Data are reported as log 2^−∆Ct^ normalized to U6 expression together with mean ± standard error of the mean (SEM) and *p*-value computed by t-test (*p* < 0.05).

As reported in Table 2 and in Figure 1, the univariate analysis showed significantly lower plasma levels of miR-133b in subjects with sarcopenia in comparison to non-sarcopenic individuals (model 1, *p* = 0.006).

**Figure 2:**
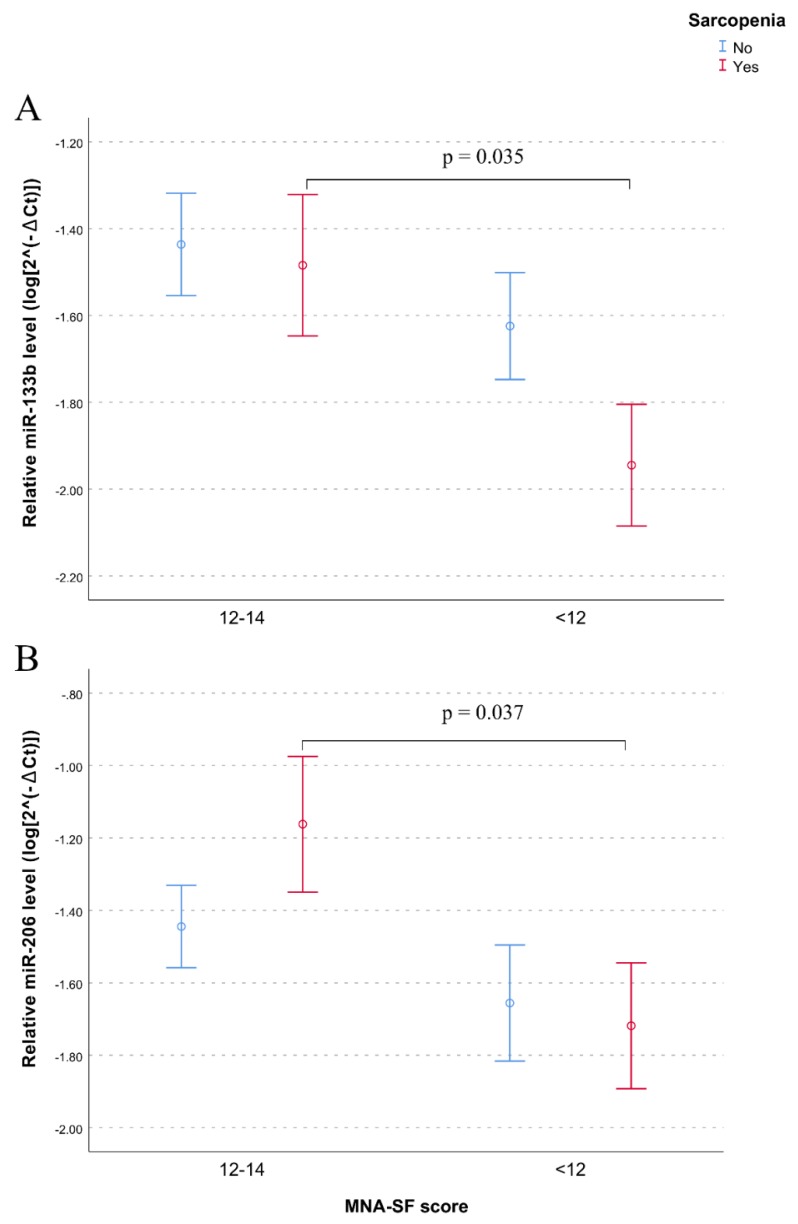
Effect of nutritional status on plasma levels of (**A**) miR-133b and (**B**) miR-206 in sarcopenic and non-sarcopenic subjects. Data are reported as log 2^−∆Ct^ normalized to U6 expression together with mean ± SEM and *p*-value computing by t-test (*p* < 0.05).

After adjusting for age and ADL, the association between miR-133b and sarcopenia remained significant (model 2, *p* = 0.037) while after additional adjustment for nutritional status, assessed by MNA-SF scores, the effect of miR-133b on sarcopenia disappeared (*p* = 0.238; model 3 in Table 2), suggesting a relationship between this miRNA and nutrition. To get more insights in this relationship we performed correlation analyses of myomiR levels with nutritional status. Data showed that lower levels of both miR-133b and miR-206 were significantly correlated with poor nutritional status assessed either by MNA-SF (miR-133b, rho = 0.193, *p* = 0.012; miR-206, rho = 0.156, *p* = 0.044) or MNA-LF (miR-133b, rho = 0.256, *p* = 0.005; miR-206, rho = 0.224, *p* = 0.014) scores. Analysis performed splitting the whole sample by sarcopenia showed that the association between lower levels of both miR-133b and miR-206 and undernutrition was statistically significant in subjects with sarcopenia (Figure 2A,B). No significant correlation for miR-133a was found.

**Figure 3:**
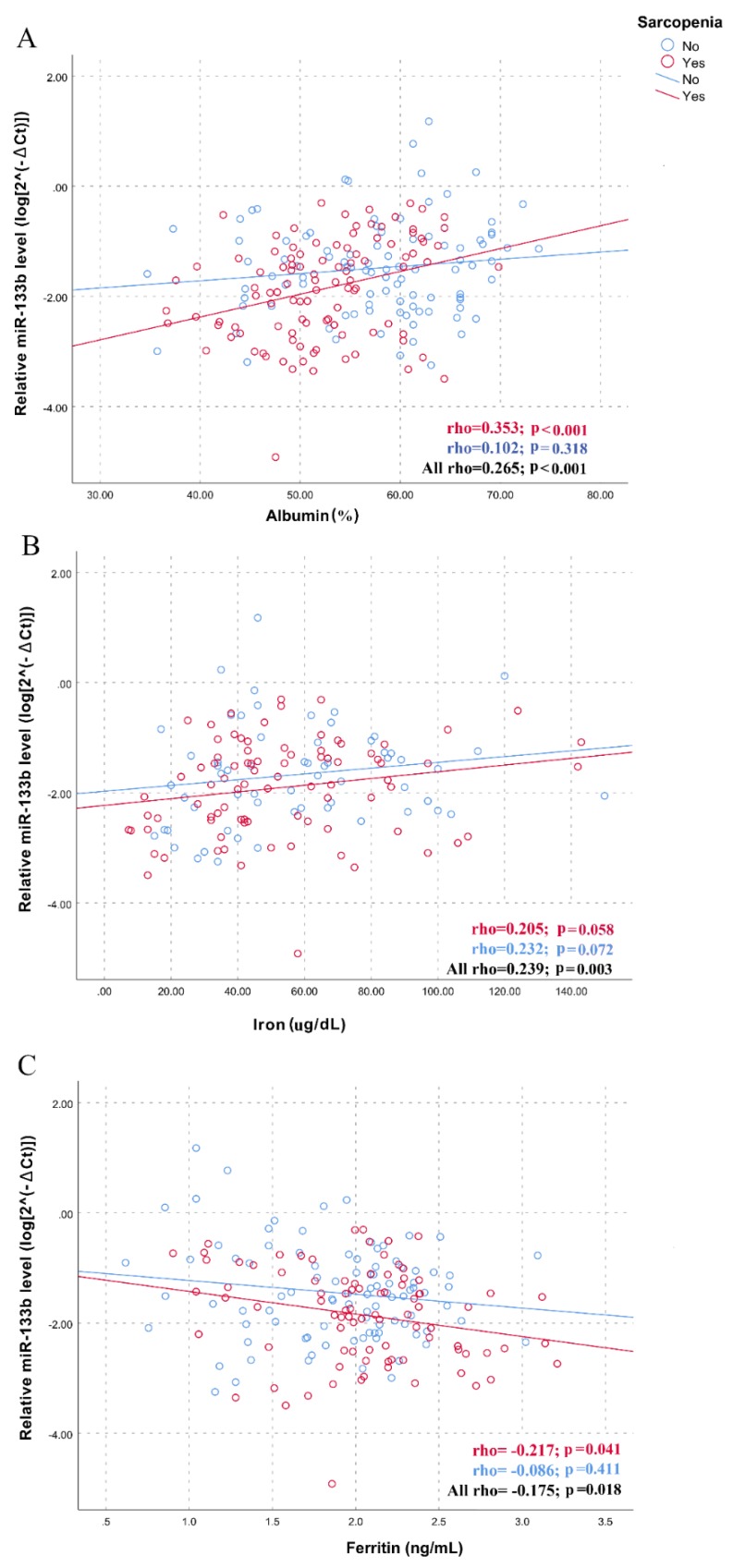
Correlations between plasma miR-133b levels and biochemical variables in sarcopenic and non-sarcopenic subjects. Scatter plots illustrate the relationship between plasma miR-133b levels and (**A**) albumin, (**B**) iron, (**C**) ferritin. Data are reported as log 2^−∆Ct^ normalized to U6 expression.

**Figure 4:**
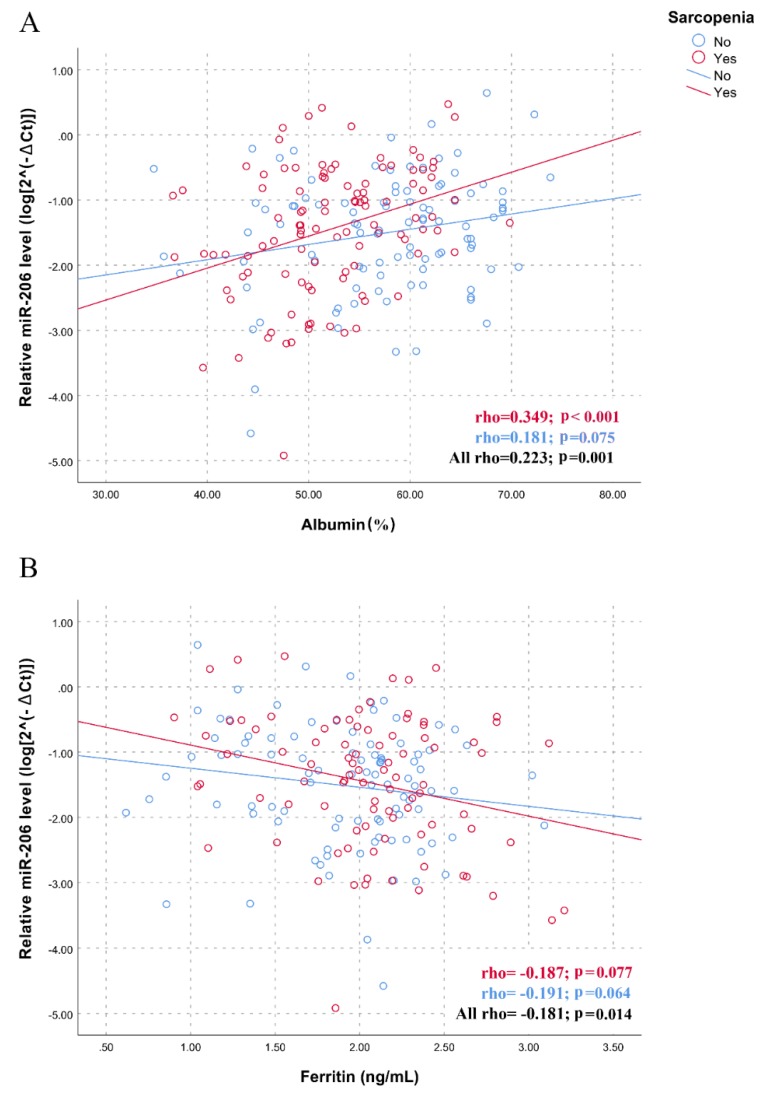
Correlations between plasma miR-206 levels and biochemical variables in sarcopenic and non-sarcopenic subjects. Scatter plots illustrate the relationship between plasma miR-206 levels and (**A**) albumin, (**B**) ferritin. Data are reported as log 2^−∆Ct^ normalized to U6 expression.

The association between myomiRs and nutritional status prompted us to assess the association between traditional blood biomarkers of malnutrition and inflammation and the levels of myomiRs, performing additional correlation analyses in the whole sample and in the two sub-groups of subjects with and without sarcopenia. Results, presented in Supplementary Materials, Table S1, show a significant positive correlation between the plasma levels of miR-133b and albumin (*p* < 0.001) and serum iron (*p* = 0.003) levels, whereas a negative correlation was observed with ferritin (*p* = 0.018), in the whole sample of participants. Furthermore, the miR-206 levels were positively and negatively correlated with albumin (*p* = 0.001) and ferritin (*p* = 0.014) levels, respectively. As shown by the scatter plots and linear regressions of Figure 3 and Figure 4, statistically more significant correlations were observed in the group of subjects with sarcopenia for both miR-133b (rho = 0.353; *p* < 0.001 for albumin; rho = 0.205; *p* = 0.058 for iron; rho= -0.217; *p* = 0.041 for ferritin; Figure 3) and for miR-206 (rho = 0.349; *p* < 0.001 for albumin; rho= -0.187; *p* = 0.077 for ferritin; Figure 4), further highlighting the potential role of nutritional status in mediating the relationship between myomiRs and sarcopenia.

## 4. Discussion

The present study was set up to assess the relationship among nutritional status, sarcopenia, and levels of myomiRs, muscle-specific miRNAs that affect skeletal muscle processes, from myogenesis to muscle homeostasis.

The analysis of the nutritional status, based on the MNA scores, showed that more than 50% of the participants to our study were malnourished or at risk of malnutrition. This high proportion is not surprising since prevalence among nursing home residents is reported to be higher compared with community-dwelling elders [40]. Anyway, consistent with several reports [41,42,43], we found that the prevalence of subjects malnourished or at risk of malnutrition was significantly higher among those with sarcopenia than those without.

The analysis of the expression profiles of the myomiRs detected in present study (miR-133a, miR-133b, and miR-206) revealed differential expression of miR-133b between subjects with and without sarcopenia, pointing to a potential role for this miRNA in the disease pathogenesis. miR-133b regulates fundamental processes of myogenesis including myoblast differentiation, regeneration, and satellite cell fate determination [26,44]. Its overexpression has been shown to occur during myogenesis [45], whereas, on the contrary, its downregulation appears to promote satellite cells quiescence, a distinctive feature of sarcopenic muscle which also shows a low regenerative capacity and an impaired differentiation potential [46,47]. Hence, these data, together with ours, support the hypothesis that downregulation of miR-133b may contribute to the decreased myogenic and regenerative capacity of muscle cells, characteristic of sarcopenia. However, when considering the nutritional status of the study population, the association between miR-133b and sarcopenia no longer remained significant. This suggests that the levels of miR-133b in plasma are correlated with the nutritional status, pointing to a mediating effect of nutrition on the relationship between miR-133b and sarcopenia. In fact, we showed that malnutrition was correlated to lower levels of miR-133b; a similar correlation was found for miR-206. Our study also showed correlations with serum markers, such as albumin and ferritin, more significant in sarcopenic with respect to non-sarcopenic individuals, thus supporting the evidence that nutritional status may mediate the relationship between sarcopenia and myomiRs.

Skeletal muscle has a significant influence on the metabolic state of the body. The age-related changes that occur within the skeletal muscle alter energy and nutrient metabolism and, in turn, this metabolic dysfunction leads to further deterioration of the skeletal muscle. Diet certainly may well be one of the factors involved in this vicious cycle, as it plays a crucial role in maintaining muscle quality and quantity, both of which have important implications for metabolic capacity and functional performance. Dietary modulation of miRNA expression has been shown to influence various diseases, such as cancer, cardiovascular disease, type 2 diabetes, and obesity [48]. Accordingly, our findings highlight a nutrient–myomiR pathway that may influence muscle myogenic capacity. In this regard, it is of interest that miR-133a/b and miR-206 appear to be directly or indirectly regulated by the mammalian target of rapamycin (mTOR) [26], the main mediator of cellular nutrient sensing and crucial regulator of skeletal myogenesis and muscle maintenance [49]. Zhang and colleagues [16] proposed a model for nutrient–mTOR–myomiR signaling in skeletal myogenesis, where the kinase-dependent mTOR pathway affects the expression of the above myomiRs through regulation of the myogenic transcription factor MyoD. According to this model, under low nutrient conditions such as amino acids and glucose starvation, mTOR is inactive and unable to induce MyoD synthesis with the consequent downregulation of miR-133a/b and miR-206. It is also well known that both sarcopenia and malnutrition are related to increased inflammation and oxidative stress [50,51,52,53]. Notably, a downregulation of miR-133b and miR-206 was observed in the muscle of patients with inflammatory myopathy [54]. Moreover, Razak and colleagues reported that treatment with tocotrienol-rich fraction (TRF), known having an antioxidant activity, increases myomiR expression in myoblasts by reducing the oxidative stress [55]. Here, significant positive and negative correlations were found between miR-133b and miR-206 levels and albumin and ferritin, respectively. Notably, decreased albumin and elevated ferritin levels are characteristic features of inflammation besides being markers of nutritional status [56,57,58]. Based on this evidence, inflammation, as well as oxidative stress, could represent factors connecting malnutrition, expression of myomiRs, and sarcopenia. To this regard, literature data report that inflammation and oxidative stress induce expression of myostatin, a member of the transforming growth factor beta (TGF-β) superfamily that inhibits skeletal muscle growth muscle mass by downregulating the expression of miR-133b and miR-206 in skeletal muscle [26,50]. In Supplementary Materials, Figure S2, we schematically illustrate the possible molecular connections in the tripartite link between poor nutrition, myomiRs and muscle wasting in the elderly. Briefly, poor nutritional status negatively influences mTOR activity, which in turn downregulates myogenic transcription factor MyoD decreasing the expression of miR-133b and miR-206. Other factors such as oxidative stress and inflammation, interrelated in a vicious circle, can influence the same axes by upregulating the levels of myostatin, a repressor of myogenesis, which, in turn, represses myomiR expression. As a consequence, the downregulation of these miRNAs contributes to muscle wasting by inducing the expression of a number of target genes, some of which have been validated and many more predicted by bioinformatics tools, that should be prioritized and checked in future investigations.

It should be pointed out that, although the sequences of mature miR-133b and miR-133a differ in only one nucleotide at the 3′ end, and that miR-206 and miR-133b constitute a bicistronic cluster on chromosome 6p12.2 (Supplementary Materials, Figure S1), in our study, miR-133a was not associated with either nutritional status or the sarcopenia, and miR-206 was not associated with sarcopenia. This is probably because these myomiRs regulate the myogenic program by activating different downstream targets, and also because their expression is regulated by different upstream signals, likely acting in a context-dependent manner [26,50]. Furthermore, many of the potential targets of myomiRs are not intimately related to myogenic processes, therefore they could be modulated by the state of other interconnected pathways. In addition, myomiRs have emerging roles in the development of a number of non-muscle cells and tissues, beyond their classification as muscle-specific factors. For instance, several reports of myomiR involvement in different types of cancers, as well as in immunological responses and inflammation processes, have emerged in the last years [50].

## 5. Conclusions

The pathogenesis of sarcopenia is multifactorial, and many of the underlying factors may not act independently in influencing the risk of disease as many of the causal pathways may overlap or interconnect. Our study supports a possible connection among nutrition, expression of miR-133b and miR-206 and age-related skeletal muscle decline; in particular, miR-133b could represent the “trait d’union” between nutritional status and susceptibility to sarcopenia.

However, the conclusions we draw should be interpreted in the context of limitations of this study. First, ours is a cross-sectional study, so results are limited to revealing associative, rather than causal, relations among nutritional status, miR-133b and miR-206 expression and muscle wasting in old age. Thus, future investigations should be carried out in order to gain insights about molecular mechanisms underlying the observed associations, exploring both downstream targets and upstream regulators of miR-133b and miR-206 in healthy and sarcopenic subjects, and to evaluate their potential as biomarkers of risk for sarcopenia and malnutrition. Second, along with diet, exercise plays an important role in maintaining muscle health because it stimulates protein synthesis. Literature data show that exercise alters the expression pattern of myomiRs, and thus, it would have been interesting to also investigate the effects of exercise on the myomiRs profile in sarcopenic patients. However, in our study, this was not possible because of the lack of information about exercise levels. Therefore, an “ad hoc” study could be designed for the purpose of accurately and comprehensively exploring this aspect which may contribute to finding preventive strategies and lifestyle changes for reducing the risk of sarcopenia.

## Figures and Tables

**Table 1:** Anthropometric and biochemical characteristics of participants with and without sarcopenia.

Variables	No Sarcopenia (*N* = 109)	Sarcopenia (*N* = 109)	*p*-Value
Age (years)	79.5 (7.3)	83.7 (6.3)	<0.001
Men (%)	39.4	33.0	0.324
HGS (kg)	22.7 (11.7)	12.4 (5.1)	<0.001
SMI (kg/m^2^)	8.5 (1.8)	6.8 (1.9)	<0.001
Gait speed (m/s)	0.69 (0.33)	0.58 (0.24)	0.094
ADL dependence (>1)	43.7%	75.9%	<0.001
MNA-SF (<12 pt)	35.4%	59.4%	0.002
MNA-LF (<24 pt)	50.0%	77.4%	0.002
Glucose (mg/dL)	104.3 (33.3)	101 (46.1)	0.555
Total protein (g/dL)	6.6 (0.5)	6.5 (0.7)	0.477
Albumin (%)	54.6 (7.9)	51.8 (6.6)	0.014
Total cholesterol (mg/dL)	169.6 (41.6)	155.4 (39.5)	0.011
Triglycerides (mg/dL)	96.6 (35.1)	85.5 (31.6)	0.025
LDL cholesterol (mg/dL)	51.1 (13.1)	49.2 (14.4)	0.315
HDL cholesterol (mg/dL)	122.3 (79.7)	116.3 (56.2)	0.530
Creatinine (mg/dL)	1.1 (0.3)	1.1 (0.5)	0.961
Uric acid (mg/dL)	4.6 (1.4)	5.6 (7.2)	0.218
Sodium (mM/L)	140.9 (2.6)	140.6 (2.5)	0.459
Potassium (mM/L)	4.4 (0.5)	4.5 (0.6)	0.728
Clorure (mM/L)	104.6 (4.5)	104 (3.7)	0.400
Calcium (mg/dL)	9.2 (0.6)	9.1 (0.6)	0.026
Phosphorus (mg/dL)	3.7 (0.6)	3.7 (1)	0.926
Magnesium (mg/dL)	1.9 (0.3)	1.9 (0.3)	0.243
Iron (μg/dL)	57.7 (29)	53.7 (28.3)	0.402
Ferritin (ng/mL) *	137.4 (177)	204.8 (279.6)	0.036
C-Reactive Protein (mg/L) *	8.9 (12.6)	17.3 (21.9)	0.040

Notes: Continuous variables are expressed as mean and standard deviations (SD), while categorical variables are expressed as percentage (%). *p* value from *t*-test for contiguous variables and from chi-squared test of association for categorical variables. * Log-transformed values. Abbreviations: SD, standard deviation; HGS: hand grip strength; SMI: skeletal muscle index; ADL: activities of daily living; MNA-SF: Mini Nutritional Assessment Short Form; MNA-LF: MNA Long Form.

**Table 2:** Effect of miR-133a, miR-133b, and miR-206 on sarcopenia according to different logistic regression models.

	Model 1		Model 2		Model 3	
	OR (95%CI)	*p*–Value	OR (95%CI)	*p*–Value	OR (95%CI)	*p*–Value
miR-133a	1.30 (0.88–1.90)	0.187	1.29 (0.85–1.97)	0.229	1.09 (0.69–1.73)	0.700
miR-133b	0.65 (0.47–0.89)	0.006	0.69 (0.49–0.97)	0.037	0.79 (0.53–1.17)	0.228
miR-206	1.06 (0.80–1.41)	0.675	1.14 (0.84–1.55)	0.413	1.20 (0.86–1.69)	0.288

Notes: Model 1: unadjusted ORs, Model 2: ORs adjusted for age and ADL, Model 3: ORs adjusted for age, ADL and MNA-SF (<12). Abbreviations: OR, odds ratio; CI, confidence interval; ADL: activities of daily living; MNA-SF: Mini Nutritional Assessment Short Form.

## Supplementary Materials

The following are available online at https://www.mdpi.com/2072-6643/12/2/297/s1, Figure S1. Genomic structure of human muscle-specific microRNAs (myomiRs), host genes and myomiRs sequence homologies. Figure S2. Schematic illustration of the hypothetical molecular connections in the tripartite link between poor nutrition, myomiRs, and muscle wasting in the elderly. Poor nutritional status negatively influences mammalian target of rapamycin (mTOR) activity, which in turn downregulates myogenic transcription factor MyoD decreasing the expression of miR-133b and miR-206, potentially leading to muscle wasting. Others factors such as oxidative stress and inflammation, interrelated in a vicious circle, can influence the same axes by upregulating the levels of myostatin, a repressor of myogenesis, which, in turn, represses myomiR expression, thus contributing to muscle wasting too. Table S1. Spearman correlations between plasma levels of miR-133a, -133b, and -206 and biochemical variables in sarcopenic and non-sarcopenic subjects.
