# Supplementary material for: Expression Patterns of Muscle-Specific miR-133b and miR-206 Correlate with Nutritional Status and Sarcopenia
Source: Nutrients. 2020 Jan 22;12(2):297. doi: 10.3390/nu12020297 (PMC7071413; doi:10.3390/nu12020297)
Supplement: Supplementary file 1 [file nutrients-12-00297-s001.zip › suppl files/Supplementary_Table S1.docx]

Table S1. Correlations between plasma levels of miR-133a, -133b and -206 and biochemical variables in sarcopenic and non-sarcopenic individuals.

| **miR-133a** | **Non sarcopenic** | | **Sarcopenic** | | **Total** | | |
| --- | --- | --- | --- | --- | --- | --- | --- |
|  | **rho** | **p-value** | **rho** | **p-value** | **rho** | **p-value** | |
| Glucose (mg/dL) | -0.016 | 0.869 | 0.117 | 0.233 | 0.034 | 0.622 | |
| Total protein (g/dL) | -0.082 | 0.505 | 0.007 | 0.947 | -0.022 | 0.779 | |
| Albumin (%) | 0.011 | 0.916 | 0.196 | 0.046 | 0.037 | 0.597 | |
| Total cholesterol(mg/dL) | 0.028 | 0.776 | -0.148 | 0.128 | -0.080 | 0.248 | |
| Tryglicerid (mg/dL) | -0.046 | 0.650 | 0.067 | 0.496 | -0.011 | 0.876 | |
| LDL cholesterol (mg/dL) | 0.058 | 0.577 | -0.157 | 0.137 | -0.052 | 0.483 | |
| HDL cholesterol (mg/dL) | -0.141 | 0.163 | -0.134 | 0.182 | -0.132 | 0.061 | |
| Creatinine (mg/dL) | -0.120 | 0.218 | -0.022 | 0.821 | -0.076 | 0.268 | |
| Uric acid (mg/dL) | -0.103 | 0.372 | -0.117 | 0.260 | -0.095 | 0.216 | |
| Sodium (mM/L) | -0.029 | 0.767 | 0.001 | 0.994 | -0.015 | 0.825 | |
| Potassium (mM/L) | 0.150 | 0.127 | 0.031 | 0.752 | 0.101 | 0.144 | |
| Clorure (mM/L) | -0.029 | 0.823 | 0.129 | 0.234 | 0.069 | 0.404 | |
| Calcium (mg/dL) | 0.052 | 0.614 | 0.065 | 0.508 | 0.036 | 0.609 | |
| Phosphorus (mg/dL) | -0.016 | 0.876 | 0.100 | 0.350 | 0.007 | 0.923 | |
| Magnesium (mg/dL) | -0.193 | 0.109 | -0.041 | 0.708 | -0.121 | 0.132 | |
| Iron (μg/dL) | 0.031 | 0.811 | 0.012 | 0.913 | 0.024 | 0.770 | |
| Ferritin (ng/mL)* | -0.087 | 0.404 | -0.173 | 0.102 | -0.129 | 0.081 | |
| C-Reactive Protein (mg/L)* | -0.013 | 0.919 | -0.042 | 0.710 | -0.033 | 0.693 | |
| **miR-133b** |  |  |  |  |  |  |  |
|  | **Non sarcopenic** | | **Sarcopenic** | | **Total** | | |
|  | **rho** | **p-value** | **rho** | **p-value** | **rho** | | **p-value** |
| Glucose (mg/dL) | -0.168 | 0.091 | -0.061 | 0.535 | -0.093 | | 0.182 |
| Total protein (g/dL) | -0.093 | 0.453 | -0.026 | 0.798 | -0.029 | | 0.717 |
| Albumin (g/dL) | 0.102 | 0.318 | 0.353 | **<0.001** | 0.265 | | **<0.001** |
| Total cholesterol (mg/dL) | 0.079 | 0.423 | 0.148 | 0.130 | 0.148 | | 0.132 |
| Tryglicerid (mg/dL) | -0.105 | 0.293 | 0.034 | 0.734 | -0.025 | | 0.723 |
| LDL cholesterol (mg/dL) | 0.064 | 0.537 | 0.122 | 0.254 | 0.131 | | 0.076 |
| HDL cholesterol (mg/dL) | 0.040 | 0.693 | 0.045 | 0.656 | 0.066 | | 0.357 |
| Creatinine (mg/dL) | -0.034 | 0.726 | -0.073 | 0.454 | -0.057 | | 0.403 |
| Uric acid (mg/dL) | -0.068 | 0.558 | -0.169 | 0.103 | -0.134 | | 0.082 |
| Sodium (mM/L) | 0.158 | 0.109 | -0.014 | 0.891 | 0.076 | | 0.275 |
| Potassium (mM/L) | 0.046 | 0.640 | -0.130 | 0.187 | -0.042 | | 0.542 |
| Clorure (mM/L) | 0.133 | 0.377 | 0.102 | 0.350 | 0.114 | | 0.168 |
| Calcium (mg/dL) | 0.077 | 0.450 | 0.096 | 0.325 | 0.111 | | 0.114 |
| Phosphorus (mg/dL) | 0.048 | 0.640 | 0.185 | 0.082 | 0.124 | | 0.092 |
| Magnesium (mg/dL) | -0.031 | 0.796 | -0.009 | 0.934 | 0.006 | | 0.940 |
| Iron (μg/dL) | 0.232 | 0.072 | 0.205 | 0.058 | 0.239 | | **0.003** |
| Ferritin (ng/mL)* | -0.086 | 0.411 | -0.217 | **0.041** | -0.175 | | **0.018** |
| C-Reactive Protein (mg/L)* | -0.075 | 0.546 | -0.101 | 0.375 | -0.110 | | 0.185 |
| **miR-206** |  |  |  |  |  |  |  |
|  | **Non sarcopenic** | | **Sarcopenic** | | **Total** | | |
|  | **rho** | **p-value** | **rho** | **p-value** | **rho** | | **p-value** |
| Glucose (mg/dL) | 0.095 | 0.337 | -0.024 | 0.805 | 0.020 | | 0.779 |
| Total protein (g/dL) | -0.188 | 0.124 | 0.147 | 0.151 | 0.023 | | 0.766 |
| Albumin (g/dL) | 0.181 | 0.075 | 0.349 | **<0.001** | 0.223 | | **0.001** |
| Total cholesterol (mg/dL) | -0.106 | 0.280 | -0.013 | 0.893 | -0.063 | | 0.359 |
| Tryglicerid (mg/dL) | -0.211 | 0.034 | -0.051 | 0.605 | -0.127 | | 0.069 |
| LDL cholesterol (mg/dL) | -0.072 | 0.490 | -0.054 | 0.613 | -0.073 | | 0.322 |
| HDL cholesterol (mg/dL) | -0.060 | 0.556 | 0.077 | 0.448 | 0.005 | | 0.945 |
| Creatinine (mg/dL) | 0.064 | 0.511 | 0.017 | 0.863 | 0.042 | | 0.540 |
| Uric acid (mg/dL) | 0.073 | 0.530 | -0.075 | 0.468 | -0.009 | | 0.912 |
| Sodium (mM/L) | -0.054 | 0.589 | 0.049 | 0.619 | -0.002 | | 0.982 |
| Potassium (mM/L) | 0.043 | 0.662 | 0.175 | 0.073 | 0.117 | | 0.090 |
| Clorure (mM/L) | -0.026 | 0.840 | 0.104 | 0.336 | 0.051 | | 0.534 |
| Calcium (mg/dL) | -0.067 | 0.509 | 0.237 | 0.014 | 0.086 | | 0.223 |
| Phosphorus (mg/dL) | 0.111 | 0.279 | 0.130 | 0.223 | 0.108 | | 0.140 |
| Magnesium (mg/dL) | -0.092 | 0.451 | 0.079 | 0.472 | -0.006 | | 0.943 |
| Iron (μg/dL) | -0.228 | 0.078 | 0.061 | 0.573 | -0.053 | | 0.524 |
| Ferritin (ng/mL)* | -0.191 | 0.064 | -0.187 | 0.077 | -0.181 | | **0.014** |
| C-Reactive Protein (mg/L)* | 0.110 | 0.374 | -0.123 | 0.276 | -0.038 | | 0.647 |
