# Supplementary figures and images for: Expression Patterns of Muscle-Specific miR-133b and miR-206 Correlate with Nutritional Status and Sarcopenia
Source: Nutrients. 2020 Jan 22;12(2):297. doi: 10.3390/nu12020297 (PMC7071413; doi:10.3390/nu12020297)

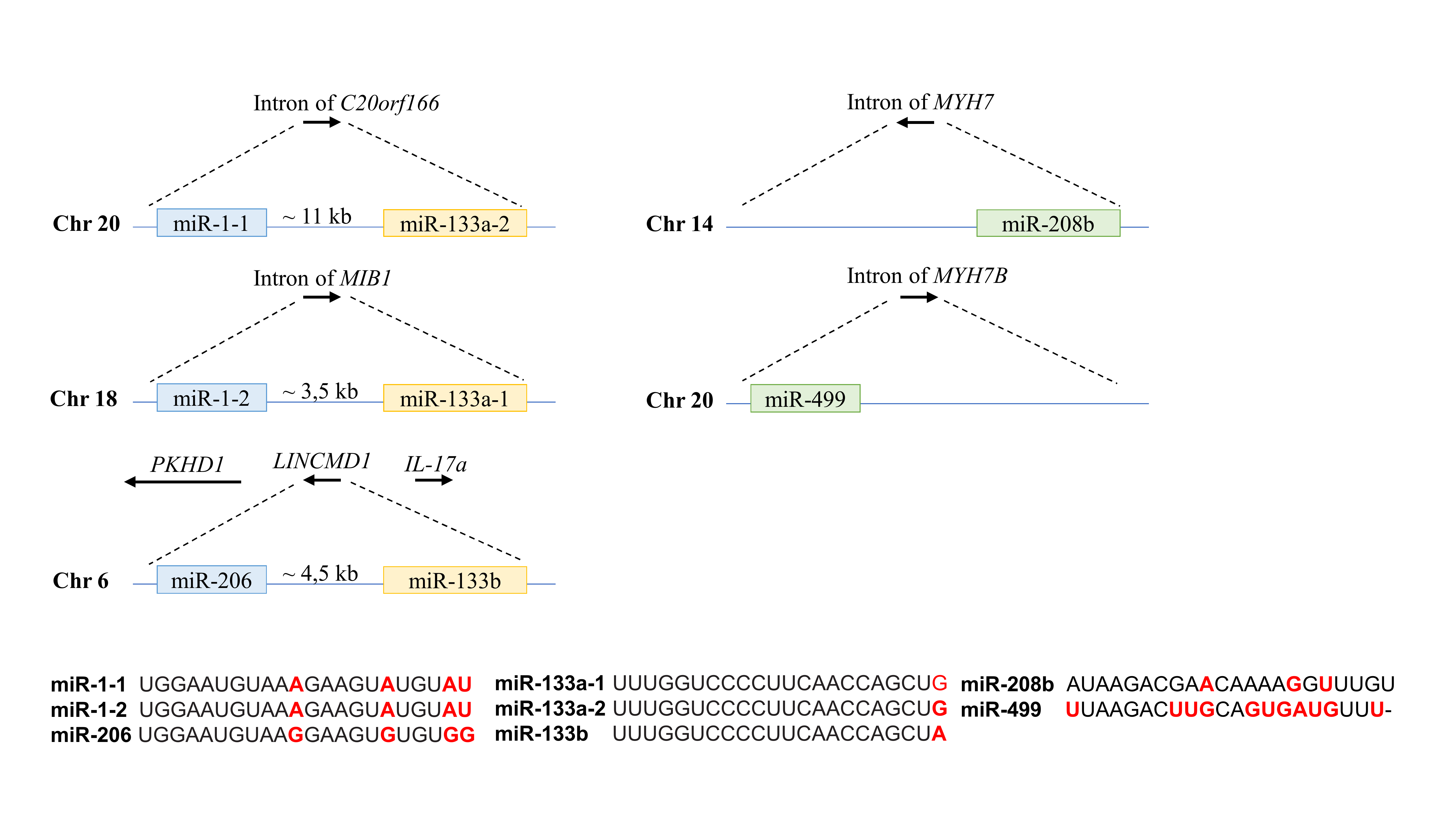

Supplement: Supplementary file 1 [file nutrients-12-00297-s001.zip › suppl files/Figure S1.tiff]

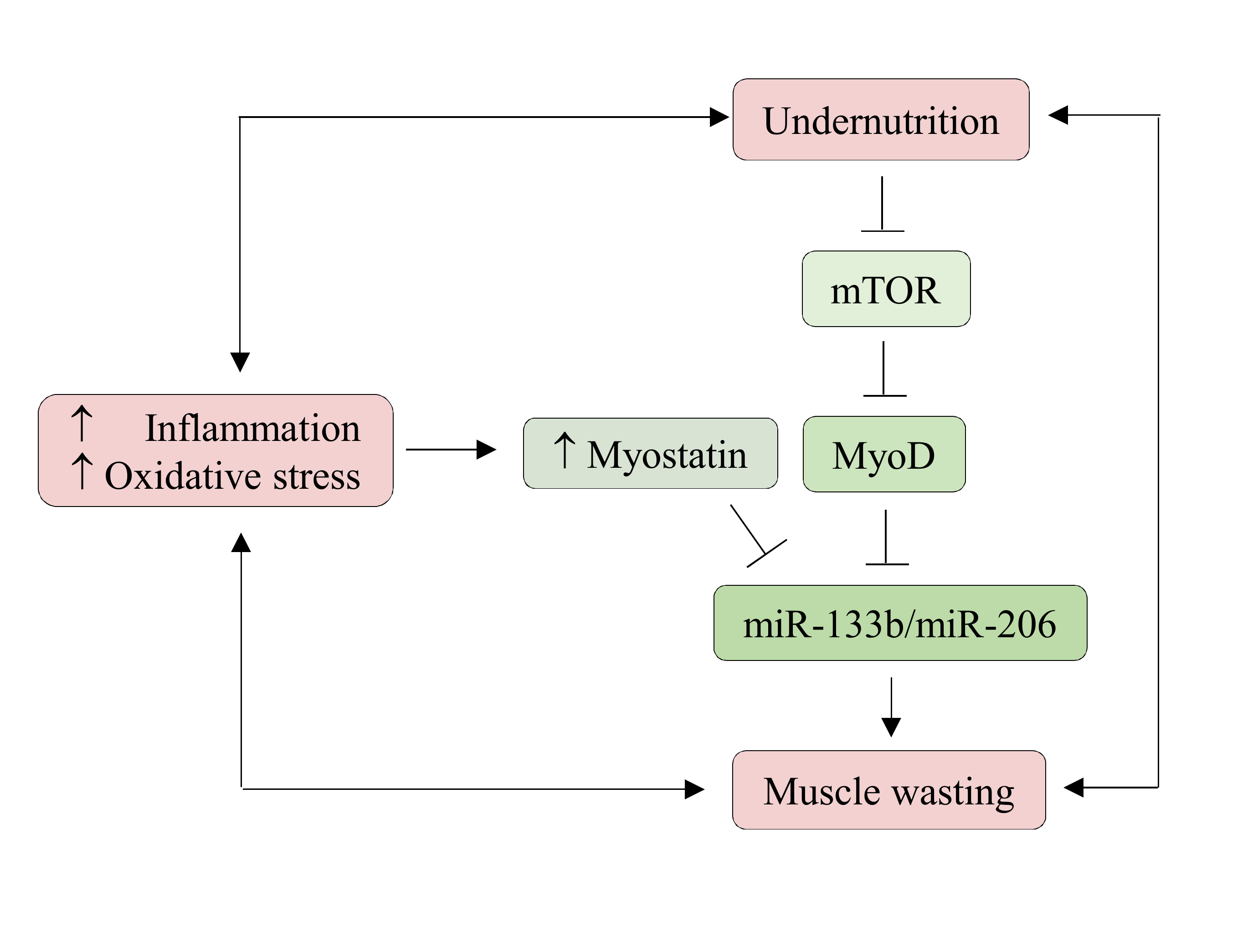

Supplement: Supplementary file 1 [file nutrients-12-00297-s001.zip › suppl files/Figure S2.tiff]
